# Artificial intelligence and the process of publishing scientific manuscripts

**DOI:** 10.1007/s00428-025-04331-y

**Published:** 2025-12-23

**Authors:** Peter Schirmacher

**Affiliations:** Institute of Pathology, Heidelberg University, Im Neuenheimer Feld 224, 69120 Heidelberg, Germany

Artificial intelligence (AI) systems are seen as a key future technology and hold great promise as powerful tools gaining increasing use also in clinical, translational and basic tissue-based research. Due to immense investments in their development and rising enthusiasm about their potential, research on AI systems and their deployment and use are rapidly progressing. In contrary, potentially adverse effects are recognized, but respective analyses and measures are lagging behind and lack comparable attention.

It is common sense among many leading experts that AI systems may not only outperform humans in certain areas, but that they may rapidly and exponentially be able to amplify their own capabilities by interconnection, autonomous decision-making, access to production chains and developing biased and hacking traits. In certain conditions, AI systems have been described to inter- and extrapolate for missing data (sometimes described as ‘hallucination’), introducing bias, developing (self)conscious-like traits and thus having the potential to deviate data generation/acquisition, decisions and execution to ‘its favour’.

In this scenario, some journals have set rules in regard to the use of AI in writing manuscripts, but the scientific publishing process appears to be a niche of lower interest; but scientific publications—if reproduced and successfully applied—represent key reference points (‘ground truth’) for further development, (medical) decision-making, funding allocation and personal careers. This holds especially true for journals, like VIAR, which publishes preferentially diagnostic and translational research data which are close to direct diagnostic application and manuscripts that directly attribute to diagnostic and clinical decision making, such as expert opinions and recommendations. AI has already been used to write manuscripts and AI systems may have been used to optimize submission strategies; AI systems have certainly been explored and used to support all stages of the publishing process from manuscript reviewing to editorial decision making up to the production process. (Pre)existing problems, such as too many low-level and even predator journals, shortage of qualified reviewers and even cheating ‘scientists’ (not a new problem, but AI may amplify their potential) may further aggravate the constellation. Recent data published in VIAR have demonstrated that the use of ChatGPT in addressing pathology-related diagnostic questions resulted in 30% incorrect referencing, of which 60% did not even exist [1]. This is especially dangerous, as these integrated ‘fake facts’ may develop their own (long) life and reality, secondary referencing and it will become almost impossible to eliminate them [2].

This scenario calls for the consideration of respective publishing measures. To my opinion, they should address all relevant decision processes in scientific publishing:

- In the case of AI-supported data generation and (meta)analyses scientific journals should check for and only accept those valid and novel scientific manuscripts that contain data generated by explainable AI, with full and free access given to all relevant data and systems information.
- The use of generative AI support in producing a manuscript (including editorials, expert opinions, recommendations and reviews) should be made transparent by mandatory and explicit statements of all authors for which supportive tasks AI has been used and that AI was not used to write the manuscript and that full personal responsibility is taken for its complete wording, references and conclusions.
- The use of generative AI systems should be banned from manuscript reviewing. Despite the rising difficulty to acquire qualified reviews it is critical that the final reviewing text is phrased by the reviewer and respective statements should be required.
- The use of AI systems should be excluded from critical editorial decision making such as selection for review, acceptance or further promotion (such as featuring, awarding)
- All journals and publishers (and the AI community) should develop and openly declare respective codes of conduct (as update to their rules of good scientific publishing) regarding the use of AI systems in the publishing process and to specifically and critically address all issues of AI in the reviewing process. It should be mandatory for publishers to provide respective governance systems, that can grow with challenge. Maybe there is a use for AI systems to detect the inappropriate use of it?
- Respective rules should become part of ‘good scientific publishing’ and journals not adhering to these principles should be excluded not only from the list of reputed scientific journals (comparable to losing the impact factor), but also from being cited or referenced, e.g. as publication achievement

We certainly need to develop sufficient expertise and provide scalable resources to intensely evaluate the (positive and negative) impact of AI systems in the process of scientific publishing and develop binding rules in order to avoid that not we, as scientific community, but AI systems may ‘decide’ how and which research is published.

Peter Schirmacher.

This opinion letter was written without any support of AI systems.
